# Effects of brain endurance training on physical and cognitive performance in athletes and physically active individuals: a systematic review

**DOI:** 10.3389/fpsyg.2026.1828644

**Published:** 2026-06-01

**Authors:** Zhang Qing, Jiang Fei, Xu Chao

**Affiliations:** 1 School of Physical Education and Health, Gannan Normal University, Ganzhou, Jiangxi, China; 2 Health Management College of Xianning Polytechnic, Xianning, Hubei, China

**Keywords:** athletic performance, brain endurance training, cognitive performance, endurance performance, mental fatigue

## Abstract

Brain endurance training (BET), a training approach that integrates cognitively demanding tasks with physical exercise, has received increasing attention in recent years. A growing body of evidence indicates that mental fatigue can impair athletic performance, executive functions, and sport-specific skills. However, a comprehensive synthesis of the overall effects of BET on both physical and cognitive performance is still lacking. Therefore, this systematic review aimed to evaluate the effects of BET on physical performance, sport-specific performance, and cognitive outcomes. A systematic search was conducted in PubMed, Web of Science, SPORTDiscus, Embase, the Cochrane Library, and Scopus. Controlled longitudinal intervention studies investigating the effects of BET interventions were included. In total, 13 controlled trials met the inclusion criteria. The intervention duration ranged from 4 to 12 weeks, with BET protocols typically combining cognitively demanding tasks (e.g., Stroop task, Go/No-Go task, AX-CPT task, and working memory paradigms) with endurance training, resistance training, or sport-specific training. The results indicate that BET consistently improves endurance-related outcomes, including time to exhaustion, time-trial performance, intermittent endurance, and sustained muscular endurance. However, these improvements are generally not accompanied by significant changes in traditional peripheral physiological markers such as maximal oxygen uptake, blood lactate concentration, or heart rate. In contrast, the effects of BET on maximal strength and repeated sprint ability appear limited or inconsistent. Improvements in sport-specific performance appear to be task-dependent and are more pronounced under conditions of mental fatigue. Most included studies reported beneficial effects of BET on cognitive performance, particularly in terms of faster reaction times and enhanced resistance to performance decrements under mentally fatiguing conditions. However, improvements in baseline cognitive performance under fresh conditions were less consistent across studies. Overall, BET may enhance endurance and cognitive performance primarily through central regulatory mechanisms rather than peripheral physiological adaptations by increasing individuals’ tolerance to mental fatigue. However, given the heterogeneity of intervention protocols and the generally moderate methodological quality of the included studies, these findings should be interpreted with caution. Future research should further investigate dose–response relationships and the long-term adaptations associated with BET.

Systematic review registation: https://inplasy.com/inplasy-2026-1-0050/, identifier INPLASY (registration number INPLASY202610050).

## 1 Introduction

In modern competitive sports environments, athletes are required to continuously engage substantial cognitive resources during both training and competition. Such prolonged or high-intensity cognitive demands may induce mental fatigue (MF), which can negatively affect athletic performance (Boksem and Tops, 2008; Martin et al., 2018). A growing body of empirical evidence indicates that even well-trained athletes may experience impairments in endurance performance, decision-making quality, sport-specific technical execution, and perceptual responses during exercise, such as increased feelings of fatigue and perceived exertion, following sustained cognitive effort (Mariano et al., 2023; Díaz-García et al., 2024a,b; Joseph et al., 2025). Given that competitive sports inherently require continuous cognitive engagement—including tactical decision-making, inhibitory control, attentional regulation, and contextual information integration—the question of how to enhance athletes’ tolerance to mental fatigue has become an important research focus in sport psychology and training science (Furley and Memmert, 2012; Ashford et al., 2021).

Because mental fatigue can substantially impair athletic performance, researchers have explored various strategies to mitigate its negative effects in recent years (Proost et al., 2022; Sun et al., 2024a; Sun et al., 2024b). Some studies suggest that mindfulness practices, meditation, and exposure to natural environments may partially counteract the detrimental effects of mental fatigue on executive control and sustained attention by facilitating attentional recovery and improving self-regulatory capacity (Azevedo et al., 2016; Cao et al., 2022; Sun et al., 2022; Cao et al., 2024; Nien et al., 2024; Park et al., 2024; Xu et al., 2024; Ding et al., 2025). These interventions are typically grounded in the attention restoration theory or mechanisms related to the redistribution of psychological resources, aiming to restore cognitive resources through brief periods of cognitive relaxation or emotional regulation (Wu et al., 2024). In addition, caffeine, a central nervous system stimulant widely used in sports settings, can reduce feelings of fatigue by antagonizing adenosine receptors and indirectly increasing dopaminergic activity, thereby enhancing alertness and reaction speed (Proost et al., 2022). Consequently, acute strategies such as caffeine ingestion may partially alleviate the immediate interference of mental fatigue on performance before competition or during match intervals. However, these approaches are primarily acute interventions, and their effects are typically transient, mainly targeting recovery or relief after fatigue has occurred. In contrast, from a training perspective, developing long-term tolerance to mental fatigue through systematic training may have greater practical relevance in competitive sports. In other words, rather than relying solely on compensatory strategies after fatigue arises, it may be more beneficial to enhance athletes’ ability to maintain performance under sustained cognitive demands through targeted training interventions.

Brain endurance training (BET) has recently emerged as a novel intervention strategy that integrates cognitively demanding tasks with physical training within the same training session, with the aim of enhancing individuals’ tolerance to mental fatigue (Marcora et al., 2014). Typical BET protocols are implemented over several weeks and incorporate cognitive tasks requiring substantial executive control—such as the Stroop task, Go/No-Go task, or working memory paradigms—before, during, or after physical training sessions. The central theoretical premise is that repeated exposure to simultaneous cognitive and physical stressors promotes adaptation to higher levels of overall effort cost, thereby enabling individuals to maintain or optimize exercise performance under conditions of mental fatigue (André et al., 2025). Recent theoretical work further suggests that BET may exert its effects by modulating neural networks associated with cognitive control, effort allocation, and endurance regulation, particularly within the prefrontal cortex and cingulate regions, thereby providing a neurophysiological basis for improvements in exercise performance (Dallaway et al., 2023; André et al., 2025).

Among athletic populations, existing empirical studies have reported generally positive intervention trends. Several experimental studies indicate that following several weeks of BET intervention, athletes may demonstrate improvements in endurance-related performance, such as prolonged time to exhaustion and enhanced time-trial performance (Dallaway et al., 2023), as well as sport-specific skills, including soccer passing and shooting accuracy, reactive agility, and padel shot performance. In addition, improvements have been observed in certain cognitive function indicators, such as reduced reaction time, enhanced inhibitory control, and increased vigilance (Díaz-García et al., 2023; Staiano et al., 2025). Moreover, BET may enhance individuals’ resistance to mental-fatigue-induced performance decrements, enabling athletes to maintain technical execution quality more effectively under mentally fatigued conditions (Staiano et al., 2023). However, the available evidence has not demonstrated consistent advantages across all performance indicators. In particular, the additional benefits of BET remain uncertain for traditional physiological markers such as heart rate responses, blood lactate concentration, and maximal oxygen uptake (de Lima-Junior et al., 2023).

Although the number of randomized controlled trials investigating BET has increased in recent years, empirical research on BET in sports settings remains at an early stage, with the overall body of literature still relatively limited and characterized by considerable heterogeneity. Substantial differences exist across studies in several key aspects, including participant characteristics (e.g., recreationally active individuals, trained endurance athletes, professional team-sport athletes, and elite youth athletes), intervention duration (typically ranging from 4 to 12 weeks), types of cognitive tasks (primarily inhibitory control and working memory paradigms), and the timing of cognitive load implementation (before, during, or after physical training). In addition, the current literature lacks a systematic integration of BET effects across multiple outcome domains, including endurance performance, sport-specific abilities, cognitive function, and psychophysiological responses. These methodological and outcome-related differences make it difficult to draw clear conclusions regarding the overall effectiveness and underlying mechanisms of BET.

To date, most publications on brain endurance training have taken the form of narrative reviews or theoretical commentaries, whereas systematic evidence synthesis evaluating the longitudinal effects of BET interventions in athletic populations remains relatively scarce. Although previous studies have reported potential benefits of BET for improving sport performance, methodological differences across studies have prevented the establishment of a coherent and comprehensive conclusion. Therefore, a structured synthesis of the available evidence is warranted to determine the extent to which BET provides additional performance benefits beyond traditional physical training, to identify the performance domains in which BET appears most effective, and to explore potential moderating factors related to intervention characteristics, such as training duration, frequency, and the timing of cognitive load integration.

Accordingly, the aim of this systematic review was to evaluate the effects of multi-week brain endurance training (BET) on physical performance, sport-specific performance, cognitive function, and perceptual responses in athletes and physically active populations. Specifically, this review sought to: (1) systematically describe the training characteristics and study designs of existing longitudinal BET interventions; (2) synthesize the direction and consistency of BET effects across different performance domains; and (3) explore the practical implications of BET for training practice, thereby providing evidence-based guidance for coaches and practitioners seeking to integrate BET into training programs.

## 2 Methods

### 2.1 Protocol and registration

This systematic review was conducted in accordance with established methodological recommendations for systematic reviews in sport and exercise science. The review protocol was prospectively registered in the International Platform of Registered Systematic Review and Meta-analysis Protocols (INPLASY) on January 15, 2026 (registration number: INPLASY202610050), prior to study screening and evidence synthesis. All procedures, including the search strategy, eligibility criteria, and data synthesis methods, were predefined to minimize potential selection bias and enhance methodological transparency.

### 2.2 Eligibility criteria (PICOS)

The eligibility criteria were defined according to the PICOS framework (Population, Intervention, Comparison, Outcomes, and Study design). Studies were included if they met the following criteria: (1) the participants were athletes, trained individuals, recreationally active individuals, or physically active individuals who engaged in regular structured exercise or sport participation, as described in the original studies. Participant classification was further interpreted according to the training and performance caliber framework proposed by McKay et al. (2022). Participants whose activity levels were limited solely to occupational, commuting, or incidental daily activities were not considered eligible for the present review (McKay et al., 2022); (2) the intervention consisted of multi-week brain endurance training (BET; ≥4 weeks) that combined cognitively demanding tasks with physical training; (3) the study included a comparison condition, such as physical training alone, habitual training, or a crossover control phase; and (4) the study reported sport-related performance outcomes, including physical performance, sport-specific performance, cognitive outcomes, perceptual measures, or psychophysiological indicators. Only longitudinal experimental study designs were considered eligible, including randomized controlled trials, non-randomized controlled trials, and crossover studies.

Studies were excluded if they: (1) investigated only acute cognitive load protocols; (2) did not implement a structured BET intervention; (3) lacked a comparison condition; (4) did not report sport-related performance outcomes; or (5) employed non-experimental study designs.

### 2.3 Search strategy

A systematic electronic search was conducted in PubMed, Web of Science, SPORTDiscus, Embase, the Cochrane Library, and Scopus from database inception to 15 January 2026 (23:00). The search strategy combined keywords related to brain endurance training, cognitive–physical combined training, athletes or physically active populations, and sport performance outcomes. Boolean operators (“AND” and “OR”) were used to combine search terms, and both controlled vocabulary and free-text terms were applied.

An example search strategy was as follows:

(“brain endurance training” OR “cognitive endurance training” OR “mental endurance training” OR “cognitive fatigue training” OR “mental fatigue training”) AND (“athletic performance” OR “sport performance” OR “physical performance” OR “technical performance” OR “decision making”) AND (athlete* OR player* OR sport*).

No language restrictions were applied. The complete search strategies for all databases are provided in the Supplementary material.

### 2.4 Study selection

The database search identified 1,780 records, with one additional record obtained from other sources. After removing 964 duplicates, 817 records remained for title and abstract screening. During this stage, studies unrelated to brain endurance training were excluded (*n* = 671), as well as studies examining only acute cognitive load (*n* = 3) and non-original articles such as reviews, or editorials (*n* = 103). Following the initial screening, 40 full-text articles were assessed for eligibility.

Full-text articles were excluded if they involved cognitive load interventions without integration with physical training or without a sustained intervention period (*n* = 23), lacked a comparison condition (*n* = 2), or did not report relevant performance outcomes (*n* = 1). Initially, 14 studies met the inclusion criteria. After methodological assessment, one study was excluded because it did not meet the predefined experimental design criteria. Consequently, 13 studies were included in the final qualitative synthesis, comprising 11 randomized controlled trials (RCTs), one quasi-randomized trial, and one single-arm cross-over study.

**Figure 1:**
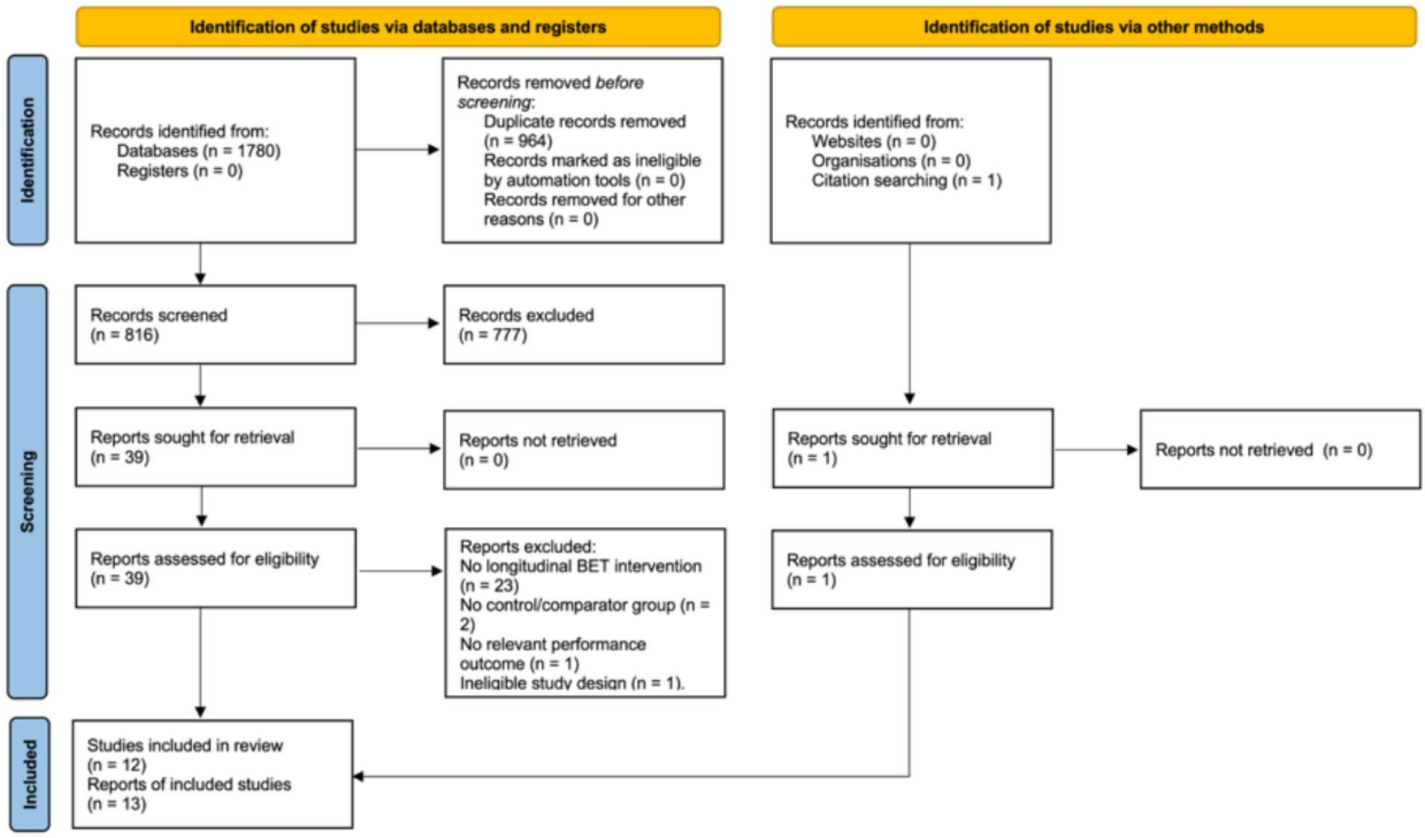
PRISMA 2020 flow diagram of the study selection process.

The study selection process is illustrated in Figure 1 (PRISMA flow diagram). All screening procedures were conducted independently by two reviewers, and disagreements were resolved through discussion with a third reviewer when necessary.

### 2.5 Data extraction

Data were extracted using a standardized data extraction form, including study characteristics, participant demographics, intervention details, comparison conditions, outcome measures, and between-group differences (Table 1).

**Table 1:** General characteristics of the included studies.

Reference	Sample	Intervention	Outcomes	Key findings
Marcora et al. (2014)	35 healthy male adults (recreationally active individuals) BET: *n* = 17; CON: *n* = 18; Male; 28 ± 6 years.	12 weeks; 3 sessions/week; 60 min/session BET: Cycling ergometer Intensity: 65% VO₂max + AX-CPT task CON: Identical handgrip training without cognitive task	VO₂max Time to exhaustion (TTE) at 80% VO₂max Rating of Perceived Exertion (RPE)	BET > CON for: Time to exhaustion (TTE) BET showed: +126% improvement in TTE Control showed: +42% improvement in TTE BET < CON for: RPE during TTE test
Dallaway et al. (2021)	36 healthy adults (recreationally active individuals); BET: *n* = 18; CON: *n* = 18; mixed sex; 20 ± 2 years.	6 weeks; 4 sessions/week; 10 min/session BET: Submaximal rhythmic handgrip (~30% MVC) + concurrent cognitive tasks (2-back, Stroop; progressive difficulty) CON: Identical handgrip training without cognitive task	Muscular endurance; Cognitive performance (working memory); Perceptual responses (mental fatigue, mental effort, RPE); Neurophysiological measures (prefrontal oxygenation); Neuromuscular activity (EMG); Cardiac responses (heart rate, HRV)	BET > CON for muscular endurance and working memory accuracy; BET = CON for MVC. BET > CON for maintenance of prefrontal cortex oxygenation; BET = CON for EMG activity, heart rate, and RPE.
Staiano et al. (2022)	22 professional male football players (professional athletes); BET: *n* = 11; CON: *n* = 11; 22.4 ± 4.3 years.	4 weeks; 4–5 sessions/week; 20–30 min cognitive task post-training BET: Standard preseason football training + post-exercise inhibitory control tasks CON: Identical preseason football training + neutral audio exposure (matched duration)	Intermittent endurance; sport-specific performance; cognitive performance; physiological and psychological measures	BET > CON for intermittent endurance and reactive agility; BET < CON for hand errors, Stroop reaction time, and PVT lapses; BET = CON for physiological measures.
De Lima-Junior et al. (2023)	45 amateur male runners (recreationally trained athletes); CT: *n* = 15; ET: *n* = 15; BET: *n* = 15; 23.4 ± 2.9 years.	12 weeks; 3 sessions/week; 20–40 min/session BET: Simultaneous endurance training (60% ΔMAV) + Stroop task ET: Endurance training only (60% ΔMAV) CT: Stroop task only	Aerobic capacity (VO₂max); Endurance performance (time-to-exhaustion); Cognitive performance (inhibitory control); Psychophysiological responses (RPE, pupil diameter)	BET = ET > CT for VO₂max and time-to-exhaustion. BET < ET and CT for RPE during TTE. BET = ET = CT for inhibitory control (reaction time and accuracy).
Staiano et al. (2023)	Study 1: 26 male trained road cyclists (trained athletes); BET: *n* = 13; CON: *n* = 13; 29 ± 5 years. Study 2: 24 male highly trained road cyclists BET: *n* = 13; CON: *n* = 11; 25 ± 4 years.	6 weeks; 5 sessions/week BET: Post-exercise inhibitory control tasks – Study 1: progressive duration (30–60 min) – Study 2: fixed duration (30 min) with progressive difficulty CON: Neutral audio exposure post-training (duration matched); physical training volume matched	Endurance performance; RPE; cognitive performance; physiological variables	BET > CON for TTE and 20-min TT; BET < CON for RPE and Stroop reaction time; BET = CON for 5-min TT and physiological measures.
Díaz-García et al. (2023)	61 amateur padel players (recreationally trained athletes); BET: *n* = 30; CON: *n* = 31; mixed sex; 28.7 ± 7.7 years.	6 weeks; 3 sessions/week; 60 min/session BET: Intermixed physical training + 5 × 4-min incongruent Stroop (20 min total) CON: Identical physical training with rest instead of Stroop during breaks	Sport-specific performance (shot speed and accuracy); Mental fatigue (VAS-MF); Cognitive performance (PVT-B); Oculomotor indices (pupil diameter, saccadic latency)	BET > CON for maintaining shot speed and accuracy under mental fatigue; BET = CON in fresh condition; BET < CON for Stroop-induced increases in VAS mental fatigue, PVT reaction time, and saccadic latency.
Dallaway et al. (2023)	24 healthy undergraduate students (recreationally active individuals); BET: *n* = 12; CON: *n* = 12; mixed sex; 20 ± 2 years.	5 weeks; 4 sessions/week BET: 20-min pre-exercise cognitive tasks (2-back, Stroop) + submaximal handgrip training (~30% MVC) CON: Identical handgrip training without cognitive task	Handgrip endurance performance; Cognitive performance (working memory); Neurophysiological and cardiac responses (prefrontal oxygenation, HR, HRV); Psychological responses (RPE, mental fatigue, motivation)	BET > CON for handgrip endurance improvement; BET > CON for prefrontal cortical oxygenation during exercise; BET = CON for working memory accuracy; BET = CON for HR, HRV, RPE, and motivation.
Díaz-García et al. (2024a,b)	91 resistance-trained athletes (trained athletes); BET: *n* = 46; CON: *n* = 45; mixed sex; 29.4 ± 10.1 years	6 weeks (+3-week follow-up); 5 sessions/week 12 min cognitive load (4 × 3-min incongruent Stroop) BET: Resistance training + Stroop CON: Identical resistance training (rest instead of cognitive task)	Strength performance (chest press, squat jump to failure); Mental fatigue (VAS-MF); Cognitive performance (PVT reaction time); Autonomic function (HRV – RMSSD)	BET > CON for chest press and squat jump under fatigue; BET < CON for Stroop-induced mental fatigue and PVT reaction time increase; BET > CON for RMSSD recovery.
Dallaway et al. (2024)	Study 1: 29 healthy undergraduate students (recreationally active individuals); BET: *n* = 14; CON: *n* = 15; 12 females, 17 males; 23 ± 5 years. Study 2: 29 healthy undergraduate student-athletes BET: *n* = 15; CON: *n* = 14; 18 females, 11 males; 21 ± 2 years.	4 weeks; 3 sessions/week; 30 min/session BET: Intermixed calisthenics + high cognitive load tasks between sets CON: Rest	Dynamic and static muscular endurance; Cognitive performance; Perceptual responses (RPE, mental fatigue)	BET > CON for dynamic exercise performance (press-ups, burpees, jump squats, leg raises) and novel task performance (mountain climbers); BET = CON for RPE.
Staiano et al. (2025)	31 professional male soccer players (professional athletes); BET: *n* = 15; CON: *n* = 16; 26.3 ± 8.4 years.	6 weeks; 3 sessions/week; 24 min cognitive load BET: Inhibitory control tasks (Flanker, Go/No-Go, AX-CPT) performed between physical training blocks CON: Identical physical training with passive rest between blocks	Soccer-specific performance (passing, shooting); Cognitive performance (PVT-B reaction time); Mental fatigue (VAS-MF)	BET > CON for passing and shooting performance under fatigue; BET = CON in the fresh condition. BET > CON for PVT-B reaction time under fatigue; BET = CON in the fresh condition.
Díaz-García et al. (2025)	24 sedentary older adults (older physically untrained adults); BET: *n* = 8; ET: *n* = 8; CON: *n* = 8; 71.4 ± 4.0 years, range 65–78.	8 weeks (+4-week follow-up); 3 sessions/week; 20 min cognitive task BET: 20-min incongruent Stroop prior to combined resistance (squats, biceps curls) and endurance walking (RPE 7–8) ET: Identical resistance + endurance training without cognitive task CON: No training	Functional capacity (6-min walk); Muscular strength (chair-stand, arm-curl); Perceptual response (RPE); Cognitive performance (PVT-B, Stroop); Assessed under fresh and fatigued conditions	BET > CON for walk distance, chair-stand, and arm-curl performance; BET > ET for chair-stand performance; BET < CON for RPE; BET > CON and ET for cognitive performance under fatigue.
Varesco et al. (2025)	19 elite youth épée fencers (elite youth athletes); BET: *n* = 11 [5F/6M]; CON: *n* = 8 [3F/5M]; 18 ± 1 years.	5 weeks; 3–4 sessions/week BET: Inhibitory control tasks implemented as dual-task or intermixed sessions before/during/after fencing training CON: Habitual fencing training only (no cognitive task)	Cognitive performance (PVT); Sport-specific performance (fencing test); Perceptual and psychological measures (VAS fatigue, NASA-TLX workload)	BET < CON for increases in PVT reaction time, PVT lapses, and perceived fatigue following cognitive fatigue. BET = CON for fencing performance (number of hits, execution time). BET = CON for NASA-TLX dimensions.
Buch et al. (2026)	18 elite orienteering athletes (13 completed) (elite athletes); 23 ± 3 years Mixed sex;	6 weeks; 3 sessions/week; ~49 min aerobic training + ~ 20–23 min cognitive task; BET group: HIT+ ~ 20 min route choice assessment (RCA); Control period: Usual physical, technical and cognitive training without BET	Cognitive performance: Stroop task (reaction time, accuracy, total correct responses); Sport-specific cognitive task (RCA response time and accuracy) Physical performance: 1000 m submaximal run; 5,000 m maximal running test; HR, blood lactate, RPE	RCA response time < CON Stroop correct responses > CON Running performance = CON HR = CON Blood lactate = CON RPE = CON

BET, brain endurance training; CON, control group; ET, endurance training; CT, cognitive training; TTE, time to exhaustion; TT, time trial; HR, heart rate; HRV, heart rate variability; RMSSD = root mean square of successive differences; RPE, rating of perceived exertion; PVT, psychomotor vigilance task; PVT-B, brief psychomotor vigilance task; VAS-MF, visual analog scale for mental fatigue; MVC, maximal voluntary contraction; EMG, electromyography; RCA, route choice assessment; AX-CPT, AX continuous performance task; AMAV, maximal aerobic velocity. “>” indicates superior outcomes compared with the comparator group; “<” indicates inferior outcomes; “=” indicates no significant between-group difference. Participant classification was interpreted according to the framework proposed by McKay et al. (2022).

Due to the heterogeneity in study populations, intervention structures, cognitive task paradigms, and outcome measures, a quantitative meta-analysis was not considered appropriate. Therefore, a direction-of-effect synthesis approach was adopted. Outcomes were categorized as BET superior to the control condition (BET > control), inferior to the control condition (BET < control), or showing no significant difference (BET = control). The results were subsequently synthesized according to the main performance domains.

### 2.6 Quality assessment

The methodological quality of the included randomized controlled trials (RCTs) was assessed using the Physiotherapy Evidence Database (PEDro) scale. If a study had been previously indexed in the PEDro database, the reported PEDro score was extracted directly. For studies not indexed in PEDro, methodological quality was independently assessed by two reviewers according to the PEDro criteria, and disagreements were resolved through discussion until consensus was reached.

Although the PEDro scale was originally developed for physiotherapy-related randomized controlled trials, it has been widely used in exercise and sport science intervention studies due to its practical applicability, interpretability, and suitability for intervention-based research. However, we acknowledge that the inclusion of non-randomized and crossover designs may limit the suitability of PEDro for all included studies.

The PEDro scale consists of 11 items (maximum score = 10) assessing internal validity and statistical reporting. Study quality was classified according to the thresholds proposed by Silverman et al. (2012): excellent (9–10), good (6–8), fair (4–5), and poor (≤3).

For non-randomized studies (e.g., crossover designs), PEDro scoring was not applied because the scale is specifically designed for randomized controlled trials. Instead, these studies were evaluated descriptively in terms of their study design and methodological characteristics. The study by Buch et al. (2026) employed a single-arm cross-over design in which participants served as their own controls during both the control and BET intervention phases (Buch et al., 2026). Therefore, PEDro scoring was not performed, and the study was described narratively. Because the study did not include randomization or blinding procedures, it may be subject to potential selection and performance bias. However, the intervention was conducted over 6 weeks in elite orienteering athletes under real training conditions, which provides relatively high ecological validity. Although the sample size was relatively small (*n* = 13 participants completed the study), the training procedures and cognitive testing protocols were clearly described and complete statistical results were reported. Consequently, the study may still provide supportive evidence regarding the potential effects of BET on cognitive performance. It should also be noted that Marcora et al. (2014) was published as a conference proceedings abstract rather than a full peer-reviewed journal article, and therefore its findings should be interpreted with caution. In addition, the PEDro scale may have limited sensitivity for assessing non-randomized and crossover study designs included in the present review.

## 3 Results

### 3.1 Study characteristics

The 13 included studies were published between 2014 and 2026 and involved a total of 514 participants. Sample sizes ranged from 18 to 91 participants. Most studies included trained or elite athletes, such as professional soccer players (Staiano et al., 2022; Staiano et al., 2025), elite orienteering athletes (Buch et al., 2026), elite youth fencers (Varesco et al., 2025), road cyclists (Staiano et al., 2023), recreational runners (de Lima-Junior et al., 2023), amateur padel players (Díaz-García et al., 2023), and resistance-trained individuals (Díaz-García et al., 2024a,b). Some studies also recruited physically active adult men (Marcora et al., 2014), recreationally trained university students (Dallaway et al., 2021; Dallaway et al., 2023; Dallaway et al., 2024), and older adults (Díaz-García et al., 2025).

Most studies employed randomized controlled trial (RCT) designs, with one quasi-randomized controlled trial (Varesco et al., 2025) and one single-arm cross-over study (Buch et al., 2026). The intervention duration ranged from 4 to 12 weeks, with training frequencies of three to five sessions per week.

The primary outcome measures included endurance performance (e.g., time to exhaustion, 5-km running performance, and cycling time-trial performance), strength or muscular endurance tasks (e.g., handgrip endurance and calisthenic exercises performed to failure), repeated sprint ability, sport-specific technical performance (e.g., passing, shooting, and fencing accuracy), and cognitive performance indicators such as reaction time and accuracy in response inhibition tasks.

### 3.2 Intervention characteristics

All included studies implemented brain endurance training (BET) protocols designed to enhance executive functions, primarily inhibitory control and working memory. Commonly used cognitive tasks included the Stroop task (incongruent or modified versions) (Staiano et al., 2022; Dallaway et al., 2023; de Lima-Junior et al., 2023; Díaz-García et al., 2023; Staiano et al., 2023; Díaz-García et al., 2024a,b; Díaz-García et al., 2025; Staiano et al., 2025), the 2-back task (Dallaway et al., 2021; Dallaway et al., 2023; Dallaway et al., 2024), the Go/No-Go task (Staiano et al., 2022; Staiano et al., 2023; Staiano et al., 2025), the Flanker task (Staiano et al., 2022; Staiano et al., 2023; Staiano et al., 2025), and the AX Continuous Performance Task (AX-CPT) (Marcora et al., 2014; Staiano et al., 2022; Staiano et al., 2023; Staiano et al., 2025). Additional tasks included the switching-stop visual task, the Multi-Source Interference Task (MSIT), and the time-load dual n-back task (Dallaway et al., 2024), as well as sport-specific cognitive training such as the route choice assessment (RCA) task used with elite orienteering athletes (Buch et al., 2026).

Across the included studies, BET was integrated with physical training using four main approaches: prior BET (cognitive tasks performed before physical training or performance testing) (Dallaway et al., 2023; Díaz-García et al., 2024a,b), post BET (cognitive tasks performed after physical training) (Staiano et al., 2022; Staiano et al., 2023), concurrent BET (cognitive tasks performed simultaneously with physical exercise) (Marcora et al., 2014; Dallaway et al., 2021; de Lima-Junior et al., 2023; Varesco et al., 2025), and intermixed BET (cognitive and physical tasks alternated within the same training session but were not performed simultaneously) (Díaz-García et al., 2023; Dallaway et al., 2024; Díaz-García et al., 2025; Staiano et al., 2025).

The duration of the cognitive tasks ranged from brief high-intensity bouts of 3–4 min embedded within training sessions to sustained high cognitive load tasks lasting 20–30 min. Across the intervention period, the total cognitive training exposure ranged approximately from 300 to 600 min. Control conditions varied across studies and included habitual training (Marcora et al., 2014; Dallaway et al., 2021; Dallaway et al., 2023; de Lima-Junior et al., 2023; Díaz-García et al., 2023; Díaz-García et al., 2024a,b; Díaz-García et al., 2025; Varesco et al., 2025), passive rest (Dallaway et al., 2024; Staiano et al., 2025), or emotionally neutral auditory stimuli (Staiano et al., 2022; Staiano et al., 2023).

Despite differences in training modalities and cognitive task types, all BET protocols aimed to induce sustained cognitive load and enhance resistance to mental fatigue.

### 3.3 Physical performance

#### 3.3.1 Endurance performance

Seven studies examined the effects of brain endurance training (BET) on endurance-related outcomes (Marcora et al., 2014; Staiano et al., 2022; Dallaway et al., 2023; de Lima-Junior et al., 2023; Staiano et al., 2023; Díaz-García et al., 2025; Buch et al., 2026). In laboratory-based endurance tests, including cycling time to exhaustion and handgrip endurance tasks, BET consistently improved performance compared with control conditions (Dallaway et al., 2023; Staiano et al., 2023). One randomized controlled trial reported that BET combined with endurance training significantly increased time to exhaustion (TTE), with a greater improvement than endurance training alone, while no between-group differences were observed for VO₂max (Marcora et al., 2014). In the same study, ratings of perceived exertion (RPE) during the exhaustion test were significantly lower in the BET group than in the control group. In cycling performance tests involving both lower- and higher-level cyclists, BET interventions increased time to exhaustion (TTE) at 65, 75, and 85% of peak power output, as well as performance in both the 5-min and 20-min time trials (Staiano et al., 2023). In addition, one study reported a significant group × time interaction, with the BET group demonstrating superior performance compared with the control group, despite no corresponding changes in traditional physiological markers such as maximal oxygen uptake, blood lactate concentration, or heart rate (Marcora et al., 2014). In contrast, the study by Buch et al. (2026) did not observe significant effects of BET on physical performance. Running performance over 1,000 m and 5,000 m, heart rate, blood lactate concentration, and ratings of perceived exertion (RPE) did not change significantly following the intervention (Buch et al., 2026).

Among trained athletes, BET was reported to improve cycling performance under mentally fatigued conditions (Staiano et al., 2023). These improvements were particularly evident when cognitively demanding tasks were performed before endurance testing, suggesting that BET may enhance athletes’ tolerance to mental fatigue (Staiano et al., 2023).

Overall, the available evidence indicates that BET may improve endurance performance primarily through central or cognitive mechanisms rather than peripheral physiological adaptations.

#### 3.3.2 Strength performance

Four studies assessed muscular endurance or strength-related outcomes (Dallaway et al., 2021; Dallaway et al., 2024; Díaz-García et al., 2024a,b; Díaz-García et al., 2025). BET interventions were associated with improvements in dynamic calisthenic exercise performance, including tasks such as push-ups, wall-sit duration, and plank duration, as well as performance in rhythmic handgrip endurance tasks. These improvements were generally moderate and were often observed under cognitively demanding conditions.

However, none of the included studies reported significant improvements in maximal strength outcomes, such as one-repetition maximum performance. Instead, the observed adaptations appeared to be limited to endurance-type muscular tasks requiring sustained effort.

Overall, the available evidence suggests that BET may improve performance in tasks involving sustained submaximal force production rather than maximal strength capacity.

#### 3.3.3 Repeated sprint ability

Currently, only one study has systematically examined the effects of brain endurance training (BET) on repeated sprint ability (RSA) in professional soccer players (Staiano et al., 2022). In this study, a 4-week BET intervention was implemented immediately after regular preseason physical training and consisted of 20–30 min of cognitively demanding tasks. The results showed that, compared with the control group, the BET group demonstrated significantly better RSA performance in directional sprint tests following the intervention. In addition, the BET group exhibited significant improvements in the 30–15 Intermittent Fitness Test (IFT speed) and in a soccer-specific reactive agility test (S-RAG completion time and number of hand errors).

Notably, the improvement in RSA was not limited to cognitive–motor dual-task or decision-making outcomes but was reflected in enhanced performance during repeated directional sprinting. This finding suggests that BET may improve athletes’ executive control under high-intensity intermittent exercise, thereby indirectly enhancing repeated sprint performance (Staiano et al., 2022).

However, as only a single study has reported these findings to date, the effects of BET on RSA require confirmation through additional high-quality randomized controlled trials.

### 3.4 Sport-specific performance

Five studies examined sport-specific performance outcomes (Staiano et al., 2022; Díaz-García et al., 2023; Staiano et al., 2025; Varesco et al., 2025; Buch et al., 2026). In soccer, BET significantly improved technical performance under mentally fatigued conditions in professional players. Specifically, following mental fatigue induced by the Stroop task, the BET group demonstrated superior passing and shooting performance compared with the control group. These improvements were reflected in higher technical success rates and a greater ability to maintain performance under fatigue, whereas no comparable changes were observed in the control group under either fresh or fatigued conditions. In addition, in tests involving multitask interference, the BET group outperformed the control group in repeated sprint ability (RSA) and reactive agility (S-RAG), suggesting that BET may enhance executive control and motor regulation under cognitive load (Staiano et al., 2022).

In padel players, although both groups showed overall improvements in stroke velocity and accuracy during the training period, the BET group demonstrated a greater ability to maintain stroke velocity and accuracy following mental fatigue induction. Moreover, the BET group exhibited smaller deteriorations in subjective mental fatigue, psychomotor vigilance task (PVT) reaction time, and eye-tracking indicators, indicating that BET may enhance the ability to sustain sport-specific technical performance under cognitively fatiguing conditions (Díaz-García et al., 2023). A sport-specific BET intervention conducted in elite orienteers reported that 6 weeks of training significantly improved decision reaction time in a route-choice cognitive task and increased the number of correct responses in the Stroop task, although no significant changes were observed in running performance or physiological measures (Buch et al., 2026).

In contrast, in fencing, although BET significantly attenuated the negative effects of cognitive fatigue on PVT reaction time and subjective fatigue, no significant differences between the BET and control groups were observed in fencing-specific performance outcomes (number of hits and execution time). This suggests that the direct effects of BET on technical execution may be limited or dependent on the sensitivity of the performance tasks and sport-specific assessment methods (Varesco et al., 2025).

Overall, current evidence suggests that BET may help enhance or preserve sport-specific performance under mentally fatigued conditions, particularly in tasks that rely heavily on executive control, attentional regulation, and rapid decision-making. However, the magnitude of these effects may vary depending on the characteristics of the sport, testing context, and the sensitivity of the performance measures used.

### 3.5 Cognitive performance

Several studies have systematically examined the effects of brain endurance training (BET) on cognitive performance, primarily focusing on executive function domains, including the psychomotor vigilance task (PVT) (Staiano et al., 2022; Díaz-García et al., 2023; Díaz-García et al., 2024a,b; Díaz-García et al., 2025; Staiano et al., 2025; Varesco et al., 2025), the Stroop task (reaction time and accuracy) (Staiano et al., 2022; de Lima-Junior et al., 2023; Staiano et al., 2023; Díaz-García et al., 2025; Buch et al., 2026), working memory (e.g., the 2-back task) (Dallaway et al., 2021; Dallaway et al., 2023), response inhibition (Dallaway et al., 2024), and memory updating (Dallaway et al., 2024).

#### 3.5.1 Reaction time and vigilance

Overall, most studies indicate that BET can improve or maintain reaction time performance, particularly under conditions of mental fatigue or high cognitive load. Several studies reported that, following mental fatigue induced by the Stroop task, the BET group showed smaller increases in PVT reaction time compared with the control group, along with a smaller increase in attentional lapses. These findings suggest that BET may enhance resistance to cognitive fatigue (Staiano et al., 2022; Díaz-García et al., 2023; Díaz-García et al., 2024a,b; Díaz-García et al., 2025).

In endurance exercise studies, the BET group demonstrated significantly faster Stroop reaction times than the control group, whereas Stroop accuracy generally did not differ between groups (Staiano et al., 2023). However, another study reported both faster reaction times and significantly improved accuracy following BET (Buch et al., 2026). Collectively, these findings suggest that BET primarily enhances information-processing speed, with comparatively smaller effects on response accuracy. In addition, some studies found that BET attenuated the increase in subjective mental fatigue (VAS-MF) and the deterioration in PVT reaction time induced by the Stroop task (Díaz-García et al., 2023; Díaz-García et al., 2024a,b).

Nevertheless, a few studies reported no significant effects of BET on baseline (fresh condition) PVT reaction time or certain cognitive outcomes (Staiano et al., 2022; Staiano et al., 2025; Varesco et al., 2025). This suggests that the benefits of BET may be more evident in maintaining cognitive performance under fatigue rather than enhancing cognition in a rested state.

#### 3.5.2 Working memory and executive function

One study reported that BET significantly improved accuracy in working memory tasks (e.g., the 2-back task) and enhanced performance in memory updating and response inhibition (Dallaway et al., 2021). BET interventions combined with physical training were also associated with faster cognitive task performance and reduced reaction time variability. However, another study found no significant improvement in working memory accuracy compared with the control group following BET (Dallaway et al., 2023).

At the neurophysiological level, some studies using near-infrared spectroscopy reported that participants in the BET group were able to better maintain prefrontal cortex oxygenation during exercise. This finding suggests that BET may support cognitive task stability by enhancing the regulation of neural resources associated with executive control (Dallaway et al., 2021; Dallaway et al., 2023).

#### 3.5.3 Resistance to cognitive fatigue

Resistance to cognitive fatigue appears to be a key mechanism underlying the cognitive benefits of BET. Several studies consistently found that, following BET interventions, participants exhibited smaller increases in subjective fatigue after cognitively demanding tasks, as well as smaller deteriorations in PVT reaction time and attentional lapses compared with control groups. In addition, some studies reported faster recovery of heart rate variability (RMSSD) after cognitive tasks in the BET group, suggesting improved autonomic regulation (Staiano et al., 2022; Díaz-García et al., 2024a,b; Varesco et al., 2025).

### 3.6 Outcomes without significant differences

It should be noted that not all cognitive outcomes responded to BET. Some studies reported no significant between-group differences in certain working memory accuracy measures or baseline PVT reaction times (Dallaway et al., 2023; de Lima-Junior et al., 2023).

### 3.7 Overall summary

Overall, current evidence indicates that the effects of BET on cognitive performance are mainly reflected in improved reaction speed, reduced negative effects of cognitive fatigue on vigilance and executive control, enhanced stability of working memory and executive functions, and better maintenance of cognitive performance under fatigue. These effects appear to be more pronounced under conditions of mental fatigue or high cognitive load, whereas improvements in baseline cognitive performance are relatively limited. This suggests that BET may primarily operate by enhancing cognitive resource regulation and fatigue resistance rather than by directly increasing baseline cognitive capacity.

### 3.8 Methodological quality

The methodological quality of the included studies was assessed using the Physiotherapy Evidence Database (PEDro) scale. Overall, the methodological quality was moderate, with PEDro scores ranging from 4 to 6. Most studies scored 5 points, while only one study achieved a score of 6, indicating an overall moderate level of methodological rigor among the included studies (Table 2).

**Table 2:** Quality assessment of included studies.

Authors (year)	Items											Score	Quality rating
	1	2	3	4	5	6	7	8	9	10	11		
Marcora et al. (2014)	Y	+	*−*	*−*	*−*	*−*	*−*	+	*−*	+	+	4	Fair
Dallaway et al. (2021)	Y	+	*−*	+	*−*	*−*	*−*	+	*−*	+	+	5	Fair
Staiano et al. (2022)	Y	+	*−*	+	*−*	*−*	*−*	+	*−*	+	+	5	Fair
de Lima-Junior et al. (2023)	Y	+	*−*	+	*−*	*−*	*−*	+	*−*	+	+	5	Fair
Staiano et al. (2023)	Y	+	*−*	+	*−*	*−*	*−*	+	*−*	+	+	5	Fair
Díaz-García et al. (2023)	Y	+	*−*	+	*−*	*−*	+	+	*−*	+	+	6	Good
Dallaway et al. (2023)	Y	+	*−*	+	*−*	*−*	*−*	+	*−*	+	+	5	Fair
Díaz-García et al. (2024a,b)	Y	+	*−*	+	*−*	*−*	*−*	+	*−*	+	+	5	Fair
Dallaway et al. (2024)	Y	+	*−*	+	*−*	*−*	*−*	+	*−*	+	+	5	Fair
Staiano et al. (2025)	Y	+	*−*	+	*−*	*−*	*−*	+	*−*	+	+	5	Fair
Díaz-García et al. (2025)	Y	+	*−*	+	*−*	*−*	*−*	+	*−*	+	+	5	Fair
Varesco et al. (2025)	Y	*−*	*−*	+	*−*	*−*	*−*	+	*−*	+	+	4	Fair

Eligibility criteria item does not contribute to total score. Items: (1) eligibility criteria; (2) randomization;(3) concealed allocation; (4) similarity at baseline; (5) subjects blinding; (6) therapists blinding; (7) assessors blinding; (8) one key outcome measured in >85% of subjects; (9) intention-to-treat analysis; (10) between-group statistical results for one key outcome; (11) measures of variability and point measures for one key outcome. “+” indicates that the criterion was met; “−” indicates that the criterion was not met. Total score out of 10 (excluding item 1).

All included studies clearly reported the eligibility criteria for participants, conducted between-group statistical comparisons, and provided point estimates with measures of variability for the reported outcomes. In addition, most studies demonstrated comparable baseline characteristics between groups and achieved relatively high follow-up completion rates, typically exceeding 85%.

However, several methodological limitations were identified. Allocation concealment was not reported in most studies, and blinding of participants, intervention providers, and outcome assessors was rarely implemented. This limitation is common in exercise training interventions, as participants can usually perceive whether they are engaging in cognitive tasks or specific training programs. Furthermore, most studies did not perform intention-to-treat analyses, which may increase the risk of bias.

Overall, despite limitations in allocation concealment and blinding procedures, the included studies demonstrate an acceptable level of methodological quality and provide moderate-quality evidence supporting the effects of brain endurance training (BET) on sport performance outcomes.

## 4 Discussion

This systematic review synthesized evidence from 13 longitudinal intervention studies, including 11 randomized controlled trials, one quasi-randomized controlled trial, and one single-arm crossover study. The review systematically evaluated the effects of brain endurance training (BET) on both physical and cognitive performance. Overall, the available evidence indicates that BET produces relatively consistent and practically meaningful improvements in endurance-related performance and executive functions, particularly under conditions of high cognitive load or mental fatigue. These findings suggest that BET may improve performance primarily through modulation of central nervous system control processes rather than through increases in physical capacity alone. Notably, the benefits of BET are more evident in the maintenance of performance under fatigued conditions, rather than in improvements during a fresh baseline state. This distinction is critical for understanding its underlying mechanisms and aligns with the persistent cognitive demands characteristic of competitive sport environments (Joshi, 2025).

### 4.1 Physical performance

#### 4.1.1 Endurance performance

The most consistent and pronounced effects of BET were observed in endurance-related outcomes. Across both laboratory and field-based endurance tests, BET was associated with improvements in time to exhaustion, running performance, cycling performance, and sustained muscular endurance (Marcora et al., 2014; de Lima-Junior et al., 2023; Staiano et al., 2023; Díaz-García et al., 2024a,b). Importantly, several studies reported improvements in endurance performance without corresponding changes in traditional physiological indicators such as maximal oxygen uptake, blood lactate concentration, or heart rate responses (Marcora et al., 2014; de Lima-Junior et al., 2023; Staiano et al., 2023). This discrepancy may reflect different mechanisms underlying endurance improvements. However, the included studies involved participants with different training and performance calibers, ranging from elite athletes to recreationally active and physically active individuals. Consequently, the magnitude and specificity of BET adaptations may differ according to baseline training status and cognitive–physiological characteristics.

Exercise performance is influenced not only by peripheral factors such as cardiorespiratory and muscular capacity but also by central regulation of perceived exertion and executive control processes (Hettinga et al., 2017; Meijen and Marcora, 2019). Through repeated exposure to high cognitive load, BET may enhance tolerance to mental fatigue, thereby reducing perceived effort during exercise and prolonging the ability to sustain physical activity. Under such conditions, endurance performance may improve even in the absence of measurable physiological adaptations. Additionally, variations in intervention protocols, training duration, and participant characteristics across studies may influence the extent to which physiological adaptations are observed. For instance, longer training periods or higher training loads may be more likely to induce cardiorespiratory adaptations, whereas short-term interventions may primarily improve performance through central neural regulation. Future research should therefore integrate neurophysiological measures, such as cerebral oxygenation or neural activity, to further clarify the mechanisms through which BET influences exercise performance.

In contrast to the findings described above, Buch et al. (2026) did not observe significant improvements in endurance performance, heart rate, blood lactate concentration, or rating of perceived exertion (RPE) following BET intervention. This discrepancy may be related to differences in the study population, as the participants in that study were national-level orienteering athletes with already high endurance capacity. In such highly trained populations, short-term BET interventions may be insufficient to elicit measurable performance improvements.

Overall, these findings support the notion that endurance performance is not solely constrained by peripheral physiological capacity but is also strongly influenced by central regulatory processes (Hettinga et al., 2017; Tornero-Aguilera et al., 2022). Repeated exposure to cognitively demanding tasks may mitigate the detrimental effects of mental fatigue on performance, enhance tolerance to sustained effort, and improve the ability to maintain task focus during prolonged exercise (Goepp et al., 2025). Thus, BET appears to primarily influence the perceptual and cognitive determinants of endurance rather than directly altering aerobic capacity. By repeatedly exposing individuals to high cognitive load, BET may enhance the efficiency and resilience of prefrontal neural networks, thereby reducing the amplification of perceived effort under mental fatigue. Consequently, BET may primarily modify the cognitive–perceptual dimension of endurance, rather than inducing peripheral physiological adaptations.

#### 4.1.2 Strength and repeated sprint ability

Compared with endurance performance, the effects of BET on maximal strength or high-intensity anaerobic tasks appear to be limited. Current evidence suggests that its benefits are primarily observed in muscular endurance or tasks requiring sustained submaximal output, whereas no significant improvements have been reported in maximal voluntary contraction (MVC) or short-duration explosive strength. This distinction indicates that the adaptations induced by BET may depend largely on the extent to which a task requires sustained executive control and regulation of effort. When performance relies predominantly on rapid neuromuscular recruitment and brief explosive output, the contribution of central cognitive processes is relatively small (Behm, 2025; Correia, 2025), thereby limiting the transfer effects of BET.

With respect to repeated sprint ability (RSA), only a single study has reported positive effects to date, which is insufficient to draw firm conclusions. This finding suggests that the influence of BET on high-intensity intermittent tasks may depend on the extent to which such tasks involve cognitive–decision demands.

### 4.2 Sport-specific performance

The effects of BET on sport-specific performance appear to be task-dependent. When performance relies heavily on executive control, decision integration, and cognitive–motor coordination (e.g., soccer passing, shooting, and reactive agility), BET may help maintain or even enhance performance under mentally fatigued conditions. In contrast, in tasks with lower demands on executive control or in highly automated technical skills (e.g., certain fencing performance indicators), BET may improve resistance to cognitive fatigue without necessarily translating into measurable improvements in sport-specific outcomes. However, when the cognitive training tasks closely align with the decision-making demands of a particular sport, BET may produce more pronounced improvements in sport-specific cognitive performance (Buch et al., 2026). Overall, the transfer effects of BET are not universal but appear to be moderated by the proportion of cognitive load inherent in the sport-specific task. When performance is primarily driven by decision quality and attentional regulation, the potential benefits of BET are likely to be greater.

### 4.3 Cognitive performance

All included studies assessed cognitive performance outcomes, with most reporting beneficial effects of BET on cognitive resilience, executive control, or resistance to cognitive fatigue. In paradigms assessing response inhibition and working memory, BET was associated with shorter reaction times, higher response accuracy, and smaller performance declines following prolonged cognitive tasks.

Importantly, after completing mentally fatiguing tasks, participants in the BET groups exhibited smaller increases in reaction time and fewer errors, indicating enhanced resistance to cognitive fatigue rather than merely improved baseline cognitive performance (Staiano et al., 2022; Dallaway et al., 2024; Staiano et al., 2025). This distinction is particularly relevant in competitive sport environments, where athletes are typically exposed to sustained cognitive demands (Klotzbier and Schott, 2024).

Furthermore, several studies reported improvements in sport-specific cognitive tasks following BET (Staiano et al., 2022; Díaz-García et al., 2023; Varesco et al., 2025), suggesting that the effects of laboratory-based executive function training may partially transfer to contexts more representative of real sporting situations (Buch et al., 2026). Overall, these findings support the central hypothesis that repeated exposure to high cognitive load can enhance executive efficiency and resistance to cognitive fatigue (Ozsoy et al., 2024).

### 4.4 Potential mechanisms

The mechanisms underlying adaptations to BET are likely mediated by central nervous system processes. Contemporary models of endurance performance emphasize the role of perceived exertion and executive control in regulating sustained exercise output. Mental fatigue is known to increase perceived exertion and impair inhibitory control, thereby limiting exercise performance.

Repeated engagement in cognitively demanding tasks may enhance neural efficiency within prefrontal networks, improve inhibitory control, and increase tolerance to cognitive stress (Nussbaumer et al., 2015; Finc et al., 2017). Such adaptations may attenuate the elevation in perceived exertion typically observed under mental fatigue, allowing athletes to sustain exercise performance for longer periods.

Importantly, most studies reported no changes in peripheral physiological indicators, further supporting the notion that BET primarily influences central regulatory processes rather than inducing adaptations in cardiorespiratory or muscular systems (de Lima-Junior et al., 2023).

Similar perspectives have also emerged from recent cognitive–physical dual-task training research. One recent study reported that combining high-intensity interval training (HIIT) with cognitive stimulation improved cognitive performance, glucose utilization, and distance covered compared with traditional HIIT alone (Guarascio et al., 2025). Although the cognitive component in that study primarily involved visuo-motor stimulation rather than the prolonged executive-function tasks emphasized in BET interventions, the findings nevertheless support the broader concept that concurrent cognitive and physical loading may influence both performance and central fatigue regulation. These findings further suggest that cognitive–physical dual-task interventions may increase metabolic and attentional demands during exercise, potentially contributing to adaptations in executive control, perceived exertion, and fatigue resistance.

### 4.5 Methodological considerations

The overall methodological quality of the included studies varied, ranging from low to moderate, with most trials receiving moderate ratings on the PEDro scale. Common limitations included the lack of allocation concealment, limited implementation of blinding procedures, and insufficient reporting of intention-to-treat analyses. In addition, sample sizes were generally small, which may reduce statistical power and limit the generalizability of the findings. Another limitation is that one of the included studies employed a single-arm crossover design without randomization (Buch et al., 2026), which may introduce selection bias.

Furthermore, considerable heterogeneity was observed across studies in terms of intervention duration, the types of cognitive tasks employed, the integration of brain endurance training with physical training (e.g., concurrent or sequential), and outcome measurements. Although the consistency of endurance-related outcomes strengthens confidence in the core findings, these variations complicate direct comparisons between studies.

Future trials should prioritize standardized reporting of cognitive training dose, progression strategies, and adherence, and should incorporate objective neurophysiological measures to better elucidate the underlying mechanisms.

### 4.6 Practical implications

From an applied perspective, brain endurance training (BET) appears to be a promising adjunct to traditional physical training, particularly for endurance sports and activities that require sustained cognitive engagement. Integrating cognitively demanding tasks into physical training sessions, either during or around training bouts, may enhance resistance to mental fatigue and improve performance under competition-like conditions.

Practitioners should ensure that both cognitive and physical loads are progressively increased and that task selection aligns with the cognitive demands of the target sport. Incorporating short, high-intensity cognitive tasks within training sessions may provide a practical strategy to enhance cognitive resilience without substantially increasing overall training time.

Based on the included studies, effective BET protocols typically implemented cognitively demanding tasks for approximately 10–30 min per session, with training frequencies ranging from three to five sessions per week over intervention periods of 4–12 weeks. Cognitive tasks were commonly integrated before, during, or immediately after physical training sessions, depending on the intended training objective. Inhibitory control tasks such as the Stroop task, Go/No-Go task, and Flanker task were among the most frequently used paradigms. Practitioners may therefore consider progressively integrating short periods of cognitively demanding tasks into sport-specific or endurance-oriented training sessions to enhance athletes’ resistance to mental fatigue.

### 4.7 Limitations

Several limitations of this review should be acknowledged. First, heterogeneity was present across studies in terms of intervention duration, types of cognitive tasks, and the integration of brain endurance training with physical training. This variability limits direct comparisons between studies and makes it difficult to identify optimal training doses or specific intervention models. Second, the included studies generally had small sample sizes, which may reduce statistical power and increase the risk of type II errors. In addition, most studies lacked long-term follow-up assessments, leaving the sustainability of BET-induced adaptations unclear. It is also noteworthy that the majority of study samples consisted of male or mixed-sex participants, which may limit the generalizability of the findings to female athletes. Furthermore, the current evidence base is dominated by a limited number of research groups, which may increase the risk of researcher allegiance bias and limit the broader generalizability of the findings. In addition, the included studies involved heterogeneous participant populations, including elite athletes, trained individuals, recreationally active participants, and physically active adults, which may influence the generalizability and interpretation of the findings. Finally, some of the included studies employed non-randomized or crossover designs, which may introduce potential bias and should therefore be interpreted with caution. In addition, one included study was published as a conference abstract and should therefore be interpreted with caution. Furthermore, although the PEDro scale has been widely used in sport and exercise science research, it may have limited sensitivity for assessing non-randomized and crossover study designs included in the present review.

Future research should conduct larger randomized controlled trials to further investigate the dose–response relationship of brain endurance training and evaluate its long-term adaptations, thereby providing more robust evidence for training practice.

## 5 Conclusion

This systematic review suggests that brain endurance training (BET) may enhance endurance-related performance and attenuate cognitive performance deterioration under mentally fatiguing conditions across different athletic populations. The most pronounced benefits were observed in endurance-related tasks and in maintaining cognitive performance under mentally fatiguing conditions. In contrast, the effects of BET on maximal strength, repeated sprint ability, and certain sport-specific performance indicators appear to be less evident.

Across most studies, traditional physiological markers showed no significant changes, suggesting that BET primarily operates through central mechanisms, likely by enhancing executive control and tolerance to cognitive fatigue rather than inducing peripheral physiological adaptations.

Although the overall methodological quality of the available evidence is moderate and sample sizes remain relatively small, the consistency of findings in endurance-related outcomes supports the potential value of BET as an adjunct training strategy. Future research should include high-quality randomized controlled trials to determine the optimal training dose, evaluate the long-term maintenance of adaptations, and clarify the applicability of BET across different sports and levels of athletic performance.

## Data Availability

The original contributions presented in the study are included in the article/Supplementary material, further inquiries can be directed to the corresponding author.
