# Supplementary material for: Effects of brain endurance training on physical and cognitive performance in athletes and physically active individuals: a systematic review
Source: Front Psychol. 2026 Jun 1;17:1828644. doi: 10.3389/fpsyg.2026.1828644 (PMC13265369; doi:10.3389/fpsyg.2026.1828644)
Supplement: Supplementary file 1 [file Data_Sheet_1.ZIP › Supplementary File 2/Search strategy by databases.pdf]

Search strategy by databases

PubMed

(("brain endurance training"[Title/Abstract] OR "cognitive endurance training"[Title/Abstract] OR "mental endurance training"[Title/Abstract] OR "cognitive fatigue training"[Title/Abstract] OR "mental fatigue training"[Title/Abstract] OR (("mental fatigue"[Title/Abstract] OR "cognitive fatigue"[Title/Abstract]) AND training[Title/Abstract]))AND("Athletic Performance"[Mesh] OR "athletic performance"[Title/Abstract] OR "sports performance"[Title/Abstract] OR "sport performance"[Title/Abstract] OR "physical performance"[Title/Abstract] OR "technical performance"[Title/Abstract])AND("Athletes"[Mesh] OR athlete*[Title/Abstract] OR player*[Title/Abstract] OR sport*[Title/Abstract]))

Cochrane Library

(brain endurance training:ti,ab,kw OR cognitive endurance training:ti,ab,kw OR mental endurance training: ti,ab,kw OR cognitive fatigue training: ti,ab,kw OR mental fatigue training:ti,ab,kw)AND(MeSH descriptor: [Athletic Performance] explode all trees OR athletic performance:ti,ab,kw OR sport performance:ti,ab,kw OR physical performance:ti,ab,kw OR technical performance:ti,ab,kw OR decision making:ti,ab,kw)AND(MeSH descriptor: [Athletes] explode all trees OR athlete:ti,ab,kw OR athletes:ti,ab,kw OR sport:ti,ab,kw OR player:ti,ab,kw OR players:ti,ab,kw)

Web of Science

TS= ("brain endurance training" OR "cognitive endurance training" OR "mental endurance training" OR "cognitive fatigue training" OR "mental fatigue training") AND TS=("athletic performance" OR "sport performance" OR "physical performance" OR "technical performance" OR "decision making" OR "reaction time") AND TS=(athlete OR athletes OR sport*)

SPORTDiscus

("brain endurance training" OR "cognitive endurance training" OR "mental endurance training" OR "cognitive fatigue training" OR "mental fatigue training") AND ("athletic performance" OR "sport performance" OR "physical performance" OR "technical performance" OR "decision making") AND (athlete* OR player* OR sport*)

Embase

("brain endurance training" OR "cognitive endurance training" OR "mental endurance training" OR "cognitive fatigue training" OR "mental fatigue training") AND ("athletic performance" OR "sport performance" OR "physical performance" OR "technical

performance" OR "decision making") AND (athlete* OR player* OR sport*)

Scopus

TITLE-ABS-KEY("brain endurance training" OR "cognitive endurance training" OR "mental endurance training" OR "cognitive fatigue training" OR "mental fatigue training")

AND

TITLE-ABS-KEY("athletic performance" OR "sport performance" OR "sports performance" OR "physical performance" OR "technical performance")

AND

TITLE-ABS-KEY(athlete* OR player* OR sport*)
