# Supplementary material for: Effects of brain endurance training on physical and cognitive performance in athletes and physically active individuals: a systematic review
Source: Front Psychol. 2026 Jun 1;17:1828644. doi: 10.3389/fpsyg.2026.1828644 (PMC13265369; doi:10.3389/fpsyg.2026.1828644)
Supplement: Supplementary file 2 [file Data_Sheet_2.ZIP › Supplementary File 3/Search strategy by databases.pdf]

**Search strategy by databases**

PubMed

(("brain endurance training"[Title/Abstract] OR "cognitive endurance training"[Title/Abstract] OR "mental endurance training"[Title/Abstract] OR "cognitive fatigue training"[Title/Abstract] OR "mental fatigue training"[Title/Abstract] OR (("mental fatigue"[Title/Abstract] OR "cognitive fatigue"[Title/Abstract]) AND training[Title/Abstract]))AND("Athletic Performance"[Mesh] OR "athletic performance"[Title/Abstract] OR "sports performance"[Title/Abstract] OR "sport performance"[Title/Abstract] OR "physical performance"[Title/Abstract] OR "technical performance"[Title/Abstract])AND("Athletes"[Mesh] OR athlete*[Title/Abstract] OR player*[Title/Abstract] OR sport*[Title/Abstract]))

Cochrane Library

(brain endurance training:ti,ab,kw OR cognitive endurance training:ti,ab,kw OR mental endurance training: ti,ab,kw OR cognitive fatigue training: ti,ab,kw OR mental fatigue training:ti,ab,kw) AND (MeSH descriptor: [Athletic Performance] explode all trees OR athletic performance:ti,ab,kw OR sport performance:ti,ab,kw OR physical performance:ti,ab,kw OR technical performance:ti,ab,kw OR decision making:ti,ab,kw)AND(MeSH descriptor: [Athletes] explode all trees OR athlete:ti,ab,kw OR athletes:ti,ab,kw OR sport:ti,ab,kw OR player:ti,ab,kw OR players:ti,ab,kw)

Web of Science

TS= ("brain endurance training" OR "cognitive endurance training" OR "mental endurance training" OR "cognitive fatigue training" OR "mental fatigue training") AND TS=("athletic performance" OR "sport performance" OR "physical performance" OR "technical performance" OR "decision making" OR "reaction time") AND TS=(athlete OR athletes OR sport*)

SPORTDiscus

TX ("brain endurance training" OR "cognitive endurance training"

OR "mental endurance training")AND TX (performance OR athlete* OR sport OR endurance OR cognition)

Embase

('brain endurance training':ti,ab,kw OR 'cognitive endurance training':ti,ab,kw OR 'mental endurance training':ti,ab,kw) AND ('athletic performance':ti,ab,kw OR 'sport performance':ti,ab,kw OR 'physical performance':ti,ab,kw OR athlete*:ti,ab,kw OR

sport*:ti,ab,kw)

Scopus

TITLE-ABS-KEY("brain endurance training" OR "cognitive endurance training" OR "mental endurance training" OR "cognitive fatigue training" OR "mental fatigue training") AND TITLE-ABS-KEY("athletic performance" OR "sport performance" OR "sports performance" OR "physical performance" OR "technical performance") AND TITLE-ABS-KEY(athlete* OR player* OR sport*)
