# Supplementary material for: Effects of brain endurance training on physical and cognitive performance in athletes and physically active individuals: a systematic review
Source: Front Psychol. 2026 Jun 1;17:1828644. doi: 10.3389/fpsyg.2026.1828644 (PMC13265369; doi:10.3389/fpsyg.2026.1828644)
Supplement: Supplementary file 2 [file Data_Sheet_2.ZIP › Supplementary File 3/Original database search histories/SPORTDiscus/Ebsco SPORTDiscus17 serch history.pdf]

正在检索：SPORTDiscus with Full Text

MyEBSCO
登录

TI (brain endurance training) OR TI (cognitive endurance training or mental endurance training or cognitive fatigue training or mental fatigue training) AND TI (performance

所有筛选器 (2) | 全文 | 同行评审 | 所有时间 | 资源类型

高级检索

自然语言检索 它是如何运作的?

结果：17

显示：20 | 相关性

已选定 0 个

1

同行评审 | 学术期刊

Brain Endurance Training Improves Physical, Cognitive, and Multitasking Performance in Professional Football Players.

作者：Staiano, Walter; Merlini, Michele; Romagnoli, Marco; +3 更多 • 位于: International Journal of ***Sports*** Physiology & ***Performance***, Dec2022, 卷 17, 期 12, 页数 1732-1740 (9p) • SPORTDiscus with Full Text

Purpose: ***Brain endurance training*** (BET)—the combination of ***physical training*** with mentally fatiguing tasks—could help ***athletes*** adapt and increase their ***performance*** during sporting competitions. Here we tested whether BET complet... 显示更多

主题：***BRAIN*** physiology; EXERCISE physiology; MOTOR ability; SOCCER; +14 更多

访问选项 | 在Scopus®中引用 36 次 | 更多类似内容 | 生成 AI 洞察

2

同行评审 | 学术期刊

Optimizing Athletic Performance Through Brain Endurance Training.

作者：Roelands, Bart; Bogataj, Špela • 位于: International Journal of ***Sports*** Physiology & ***Performance***, Oct2024, 卷 19, 期 10, 页数 973-974 (2p) • SPORTDiscus with Full Text

The authors discuss the role of ***brain endurance training*** (BET), an innovative ***training*** methodology that integrates both ***physical*** and ***cognitive*** components within a single ***training*** session, in optimizing ***athletic performance***. Topics incl... 显示更多

主题：***BRAIN*** physiology; QUALITY of life; ***ATHLETIC*** ability; ***ENDURANCE sports training***; +4 更多

访问选项 | 在Scopus®中引用 4 次 | 更多类似内容 | 生成 AI 洞察

试用水印
