# Supplementary material for: Effects of brain endurance training on physical and cognitive performance in athletes and physically active individuals: a systematic review
Source: Front Psychol. 2026 Jun 1;17:1828644. doi: 10.3389/fpsyg.2026.1828644 (PMC13265369; doi:10.3389/fpsyg.2026.1828644)
Supplement: Supplementary file 2 [file Data_Sheet_2.ZIP › Supplementary File 3/Original database search histories/Scopus/Scopus12serch history.pdf]

机构信息

Scopus

检索 来源出版物 SciVal

高级查询

检索范围: 论文标题、摘要、关键词 | 关键字检索 *: brain endurance training OR cognitive endurance training OR mental endurance tra

AND

检索范围: 论文标题、摘要、关键词 | 关键字检索: performance OR athletic performance OR sport performance OR physical performa

AND

检索范围: 论文标题、摘要、关键词 | 关键字检索: athlete OR sport OR player

保存检索

设置检索通知

添加检索字段

重置 检索

文献 预印本 测试版 辅助文献

找到 12 篇文献

分析结果

全部 Export 下载 引文概览 更多 显示所有摘要 排序依据 日期（最近）

细化搜索

在搜索结果内搜索

筛选器

年份

范围 单个

从 – 到

作者姓名

学科类别

Medicine 9

Health Professions 5

1 Article • 开放获取
Effects of 5-Week Brain Endurance Training on Fatigue and Performance in Elite Youth Epée Fencers
Varesco, G., Staiano, W., Bracco, M., ... Doron, J., Jubeau, M.
International Journal of Sports Physiology and Performance, 2025, 20(7), 页 979–985
查看摘要 View at Publisher 相关文献
1 引文

2 Review • 开放获取
The Effect of Transcranial Direct Current Stimulation on Basketball Performance—A Scoping Review
Chmiel, J., Buryta, R.
Journal of Clinical Medicine, 2025, 14(10), 3354
查看摘要 View at Publisher 相关文献
0 引文

3 Article • 开放获取
Brain endurance training improves soccer-specific technical skills and cognitive performance in fatigued professional soccer players
Staiano, W., Díaz-García, J., García-Calvo, T., Ring, C.
Journal of Science and Medicine in Sport, 2025, 28(1), 页 69–76
查看摘要 View at Publisher 相关文献
7 引文

Scopus AI New 利用近期工作概要查找研究参考文献。 试用 Scopus AI

试用水印
