# Supplementary material for: Effects of brain endurance training on physical and cognitive performance in athletes and physically active individuals: a systematic review
Source: Front Psychol. 2026 Jun 1;17:1828644. doi: 10.3389/fpsyg.2026.1828644 (PMC13265369; doi:10.3389/fpsyg.2026.1828644)
Supplement: Supplementary file 2 [file Data_Sheet_2.ZIP › Supplementary File 3/Original database search histories/WOS/WOS1121.pdf]

Web of Science Search Strategy (v0.1)

Database: Web of Science Core Collection

Entitlements:

- WOS.IC: 2009 to 2014
- WOS.CCR: 2009 to 2014
- WOS.SCI: 1990 to 2026
- WOS.AHCI: 1990 to 2026
- WOS.BHCI: 2009 to 2015
- WOS.BSCI: 2009 to 2014
- WOS.ESCI: 2015 to 2026
- WOS.ISTP: 1990 to 2026
- WOS.SSCI: 1990 to 2026
- WOS.ISSHP: 1990 to 2026

Searches:

1: brain endurance training (Title) OR cognitive endurance training (All Fields) OR mental endurance training (All Fields) OR cognitive fatigue training (All Fields) OR mental fatigue training (All Fields) Date Run: Fri Jan 16 2026 00:22:[illegible]
5050

2: athletic performance (Title) OR sport performance (All Fields) OR physical performance (All Fields) OR technical performance (All Fields) OR decision making (All Fields) OR reaction time (All Fields) Date Run: Fri Jan 16 2026 00:24:04 GMT+[illegible]

3: athlete (Title) OR Athletes (All Fields) OR sport* (All Fields) Date Run: Fri Jan 16 2026 00:24:49 GMT+0800 (N-Výh QÆeö•ô) Results: 1633273

4: #1 AND #2 AND #3 Date Run: Fri Jan 16 2026 00:27:[illegible]
1121
