# Supplementary material for: Effects of brain endurance training on physical and cognitive performance in athletes and physically active individuals: a systematic review
Source: Front Psychol. 2026 Jun 1;17:1828644. doi: 10.3389/fpsyg.2026.1828644 (PMC13265369; doi:10.3389/fpsyg.2026.1828644)
Supplement: Supplementary file 2 [file Data_Sheet_2.ZIP › Supplementary File 3/Original database search histories/Embase/Embase197.html]

Exported HTML | Embase Embase

# Embase session results (15 Jan 2026)

| No. | Query | Results |
| --- | --- | --- |
| #10 | #3 AND #6 AND #9 | 139 |
| #9 | #7 OR #8 | 233997 |
| #8 | athlete\*:ti,ab,kw OR sport\*:ti,ab,kw | 220697 |
| #7 | athlete | 91860 |
| #6 | #4 OR #5 | 477034 |
| #5 | 'athletic performance':ti,ab,kw OR 'sport performance':ti,ab,kw OR 'physical performance':ti,ab,kw OR 'technical performance':ti,ab,kw OR 'decision making':ti,ab,kw OR 'reaction time':ti,ab,kw | 456745 |
| #4 | athletic AND performance | 29860 |
| #3 | #1 OR #2 | 2325 |
| #2 | 'brain endurance training':ti,ab,kw OR 'cognitive endurance training':ti,ab,kw OR 'mental endurance training':ti,ab,kw OR 'cognitive fatigue training':ti,ab,kw OR 'mental fatigue training':ti,ab,kw OR (('mental fatigue':ti,ab,kw OR 'cognitive fatigue':ti,ab,kw) AND training:ti,ab,kw) | 410 |
| #1 | 'brain endurance training' OR (('brain'/exp OR brain) AND ('endurance'/exp OR endurance) AND ('training'/exp OR training)) | 1944 |

Copyright © 2026 Elsevier Limited except certain content provided by third parties.

Embase is a trade mark of Elsevier Life Sciences IP Limited.
